# Habitat preferences and genetic diversity of the amphipod *Gammarus roeselii* across the Eastern Alps and western Pannonian Basin

**DOI:** 10.1038/s41598-026-39958-7

**Published:** 2026-02-13

**Authors:** Špela Di Batista Borko, Jacqueline Grimm, Christoph Hahn, Péter Takács, Anna-Maria Greilberger, Stephan Koblmüller, Kristina M. Sefc

**Affiliations:** 1 Institute of Biology, University of Graz, Graz, Austria; 2 HUN-REN Balaton Limnological Research Institute, Tihany, Hungary

**Keywords:** Ecology, Ecology, Evolution, Genetics

## Abstract

Freshwater amphipods often exhibit cryptic diversity and are undergoing range shifts driven by environmental changes. *Gammarus roeselii*, a species inhabiting streams, rivers and lakes across Europe, diversified into ancient genetic lineages in the Balkan Peninsula and the Pannonian Basin, with only one lineage colonizing Central and Western Europe after the ice ages. We investigated the distribution and genetic diversity of *G. roeselii* by sampling over 1,000 sites across the eastern Alpine region and the Western Pannonian Basin. Cytochrome oxidase I barcoding assigned all sequenced *G. roeselii* (528 individuals from 174 sites) to the Central-Western European lineage. The occurrence of *G. roeselii* was associated with low elevation, high summer temperature and gentle stream and river slopes and was biased towards downstream reaches and rivers with large drainage sizes. Its distribution partially overlapped with *G. fossarum*, which predominates in cooler, faster-flowing streams. Species distribution modelling under future climate scenarios predicted a range expansion of *G. roeselii* into current *G. fossarum* habitat. Genetic diversity patterns are consistent with longstanding stable populations in the Southwestern Pannonian Basin, a post-glacial range expansion across alpine forelands, and recent colonization of some alpine valleys.

**Supplementary Information:**

The online version contains supplementary material available at 10.1038/s41598-026-39958-7.

## Introduction

Amphipods constitute an important part of freshwater ecosystems in terms of their biomass and ecosystem function^1,2^. The nominal diversity of freshwater epigean amphipods in Europe is highest in Southern and South-Eastern Europe, while it is low in Central and Western Europe^3^. In Central-Western Europe, *Gammarus fossarum*,* G. pulex*, and *G. roeselii* are the most abundant species in rivers and streams, and *G. lacustris* constitutes the native amphipod fauna of lakes^4^. *Gammarus fossarum* and *G. roeselii* exhibit high levels of cryptic diversity, a common phenomenon within amphipods^5–9^. In recent decades, the native European amphipod fauna was highly influenced by human-mediated colonisations of alien amphipod species^10^. In many areas, invaders have partially or completely replaced native amphipod species^11–14^. Many invasive species originate from the Ponto-Caspian region. However, among native species, *G. roeselii* is also expanding its range and establishing new populations in adjacent areas, where it has a strong influence on local invertebrate communities^13,15^.

*G. roeselii* Gervais, 1835 is widely distributed in rivers and lakes of the Balkan Peninsula, the Pannonian Basin, and Central-Western Europe^4^. It is a species complex that consists of 13 known Molecular Operational Taxonomic Units (MOTUs), some of which are narrowly distributed^6,16^. Most of the MOTU diversity occurs in South-Eastern Europe, with only two non-south-eastern MOTUs present in the Pannonian Basin and Central-Western Europe: a widespread MOTU C, and MOTU O, the latter found at only one location^6,16^. The species complex originated in the Miocene and diversified predominantly in the Balkan Peninsula, from where it dispersed into Eastern and Central Europe in the Pliocene. After the last glacial maximum, the colonisation of the rest of Europe began in the Pannonian Basin^16^, similarly as in several other freshwater taxa^17–20^. *G. roeselii* is usually considered native in Eastern and Central European rivers, but currently appears to be expanding its geographic and ecological distribution^4,6,16,21^. Csapó et al.^16^ analysed the genetic structure within the Central-Western European MOTU C, and showed that it consists of 11 BOLD BINs based on COI barcode sequences (note that two of the BINs recognised in their study^16^, namely ADD4052 and ADD6100, were merged with AAY1309 in the newest BOLD delineation, and are not valid anymore). The BINs within MOTU C represent the mitochondrial structure and are not well reflected in nuclear sequence divergence^16^. All known BINs of MOTU C are present in the Pannonian Basin, with only one valid BIN, AAY1309, having colonized all of Central-Western Europe. This suggests that the Pannonian Basin was an extra-Mediterranean glacial refugium, while the occurrence of *G. roeselii* in Central-Western Europe follows post-glacial demographic and spatial expansion^16^. Furthermore, documented recent range expansions at the edge of the distribution in the past 50 years and several cases of dislocated haplotypes (geographically isolated from the rest of the haplotype range) support the hypothesis of human - and global warming - induced dispersal^13,16,22^.

Across its distribution, *G. roeselii* occupies various types of epigean habitats such as lakes, rivers, streams, and different artificial water bodies^6^. Outside the Balkans, the species is primarily found in the lower reaches of rivers and artificial channels^16^, although it also occurs in lakes, where it has sometimes displaced *G. lacustris*^13^. It shows high phenotypic plasticity and tolerance to various biotic and abiotic disturbances, which makes it a successful coloniser^21–23^. In the middle and lower sections of the rivers it may outcompete other native amphipod species as it is more resilient to pollution and habitat degradation^24,25^, has better antipredatory strategies (dorsal spines and antipredatory behaviour, such as night drift)^23,26^, and is less sensitive to high temperatures and low oxygen^27,28^. However, it often coexists with *G. pulex* and *G. fossarum*^26,29^. Noteworthy, despite its competitive advantages over other native amphipods, *G. roeselii* is currently being replaced by more aggressive invaders such as *Dikerogammarus villosus*, especially in large rivers and lakes^12,30^.

Rising water temperatures associated with climate change and the impoundment of rivers and streams increase the competitiveness of G. *roeselii* with species inhabiting fast-flowing, cool streams such as *G. fossarum*. Detailed comparisons between the two species revealed different thermal optima for maximum fecundity (12.1 °C for *G. fossarum* and 16.3 °C for *G. roeselii*)^27,31^. At temperatures below 12 °C, *G. fossarum* reaches sexual maturity faster, has a shorter brood development and achieves a higher reproductive success than *G. roeselii*^32^. In contrast, higher temperatures favor *G. roeselii*, which has an optimal temperature range between 16 °C and 20°C^27,31,33^.

The present study investigates the distribution, habitat preferences and genetic diversity of *G. roeselii* in the Eastern Alpine region and the Western Pannonian Basin, specifically across Austria and in adjacent regions of neighbouring eastern countries (Hungary, Slovenia and Slovakia). Austria is located in the eastern part of the European Alps. A large part of the country is characterized by alpine and alpine foreland landscapes. The lowlands of eastern Austria belong to the Pannonian Basin (also known as Carpathian Basin), which extends eastwards to include most of Hungary and parts of Slovenia, Croatia, Serbia and Slovakia. *Gammarus roeselii* is known to inhabit the lowland water bodies of the Pannonian Basin in Austria and other countries^34–36^, but its distribution in the Austrian Alps and the alpine forelands remained largely unexplored. We collected presence/absence data in lotic waterbodies across Austria to investigate the habitat preferences of *G. roeselii*, assess its ecological differentiation from *G. fossarum* and predict its future distribution under different climate change scenarios. Furthermore, the study region lies at the border between the region where *G. roeselii* display high genetic diversity, i.e., the Balkan Peninsula and southern Pannonian Basin, and the genetically uniform Central-Western Europe populations. While the alpine parts of our study region were glaciated during the last ice age, the alpine forelands and the Pannonian Basin were ice-free, and the lowlands within our study region may have been part of the Pannonian ice-age refugium proposed by Csapo et al.^16^. Moreover, the eastern Alps have served as glacial refugia for a range of species (butterflies^37,38^; snails^39,40^; alpine plants^41^; trees^42^; reviewed in Schmitt and Varga^17)^, and *G. roeselii* may also have survived there during glaciation. Using cytochrome C oxidase subunit I (COI) gene sequences ^43^,we examined the distribution of genetic diversity of *G. roeselii* in the study region and explored its implications for the demographic history of the species in various parts of the study region.

## Results

### Distribution and habitat preferences

**Fig. 1:**
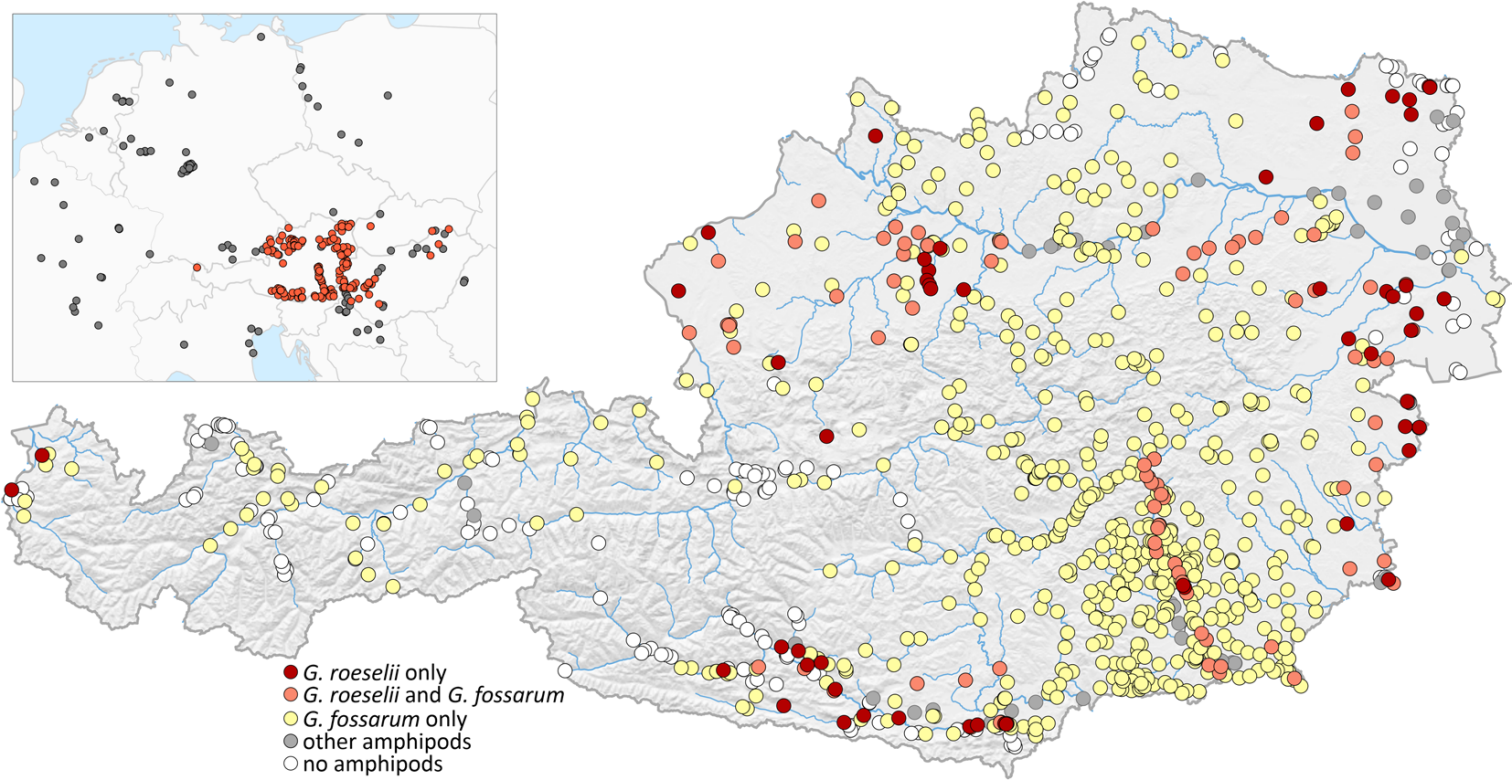
Sampling sites in Austria, indicating presence of *G. roeselii* and/or *G. fossarum*, sites with other amphipod species, and sites where we did not find any amphipods. Inset: Sites with available COI sequences of *G. roeselii* in Europe. Red: own samples; dark grey: Genbank and BOLD. Map was generated using R 4.4.3.

We detected *G. roeselii* at 139 of the 1003 sites examined across Austria. *Gammarus roeselii* and *G. fossarum* occurred syntopically at 85 of those sites, and *G. fossarum* (but not *G. roeselii*) was found at an additional 631 sites. Other amphipod species were found at 61 sites, while no amphipods were found at 172 sites (Fig. 1, Supplementary Table S1). *Gammarus roeselii* was mainly limited to the eastern lowlands and pre-alpine areas of Austria: in the Northern Alpine and Carpathian Foreland, in the Pannonian Basin and the South-Eastern Alpine Foreland (Fig. 1). The species is mostly absent from the alpine mountain regions, except for an area of the river Drava Basin in the Southern Alps (Fig. 1). We also detected *G. roeselii* in western Austria, near Lake Constance. In contrast, *G. fossarum* is widespread and was found both in lowlands and alpine mountain regions, although it was missing from many waterbodies in the central alpine region (Fig. 1). Both *G. roeselii* and *G. fossarum* were absent from sites in the eastern-most lowland regions, where suitable streams are scarce and ditches and ponds are inhabited by *Synurella* sp. (unpublished data).

**Fig. 2:**
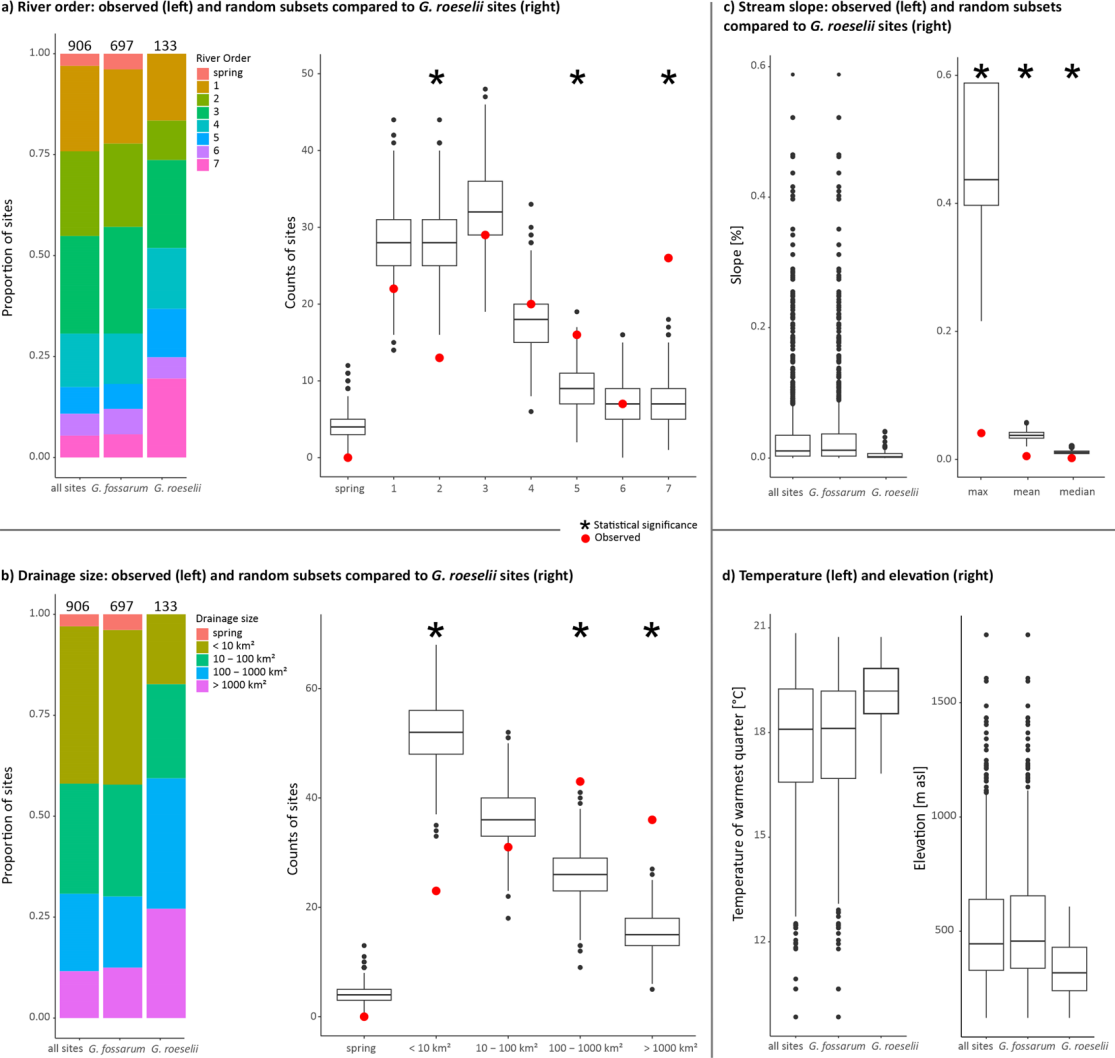
Habitat characteristics of *G. roeselii* and *G. fossarum* sites in the Austrian part of the Danube Basin. (**a**) River order. On the left, stacked bar plots show the frequencies of river order categories for all sites, *G. fossarum* sites and *G. roeselii* sites. On the right, boxplots show counts of river order categories in 1000 random subsets of sites (*n* = 133), compared to counts of actual *G. roeselii* sites (red dots). Asterisks indicate significant deviations of observed counts from random expectations (p-value < 0.025). (**b**) Drainage size. Plots are organised as in a). (**c**) Stream slope. On the left, boxplots show the distributions of stream slopes at all sites, *G. fossarum* sites and *G. roeselii* sites. On the right, boxplots show the distributions of summary statistics (maximum, mean and median) of 1000 random subsets of sites (*n* = 133), compared to values of actual *G. roeselii* sites (red dots). Asterisks indicate significant deviations of observed values from random expectations (p-value < 0.025). (**d**) Distributions of temperature of warmest quarter (left) and elevation (right) at all sites, *G. fossarum* sites and *G. roeselii* sites.

Based on 906 sites in lotic habitats of the Danube Basin, we inferred the type of habitat in which *G. roeselii* occurs and the extent to which its preferences overlap with the distribution of *G. fossarum*. *Gammarus roeselii* was generally found at lower elevations than *G.* *fossarum* and in areas with a higher mean temperature during the warmest quarter (Fig. 2d). The sampling sites encompassed a wide range of river orders (from 1 to 7 as well as springs) and drainage sizes (< 10 to > 1000 km^2^). *Gammarus roeselii* was associated with larger rivers and larger drainage sizes, whereas it was absent from springs. *Gammarus roeselii* sites were significantly underrepresented in lower order streams with smaller drainage areas. The pattern changed at river orders 3 to 4 and drainage sizes of 10–100 km^2^, where *G. roeselii* sites began to be overrepresented compared to random expectations (Fig. 2a and b; Supplementary Table S3). Stream slopes at the sampling sites ranged from zero to 59%, and *G. roeselii* only occurred at sites with gentler slopes. Summary statistics of slopes at *G. roeselii* sites (mean 0.5%, median 0.2%, max 4.1%) were statistically significantly lower than expected by chance, i.e., compared to random subsets of sampling sites (Fig. 2c; Supplementary Table S3). Generally, stream slope correlates with river order and drainage size (that is, smaller streams often have steeper slopes). However, the slopes of *G. roeselii* sites did not differ by river order and drainage category, and among the sampled streams with low river order and small drainage size, *G. roeselii* occurred only in those with gentle slopes (Fig. S1).

While *G. roeselii* was associated with either larger rivers or small streams with gentler slopes, *G. fossarum* was found in diverse lotic habitats, ranging from springs to higher-order rivers (Fig. 2). The frequencies of “presence” sites per drainage size category and per river order level differed significantly between *G. roeselii* and *G. fossarum* (drainage sizes: χ^2^ = 48.136, p-value = 4.9e-04; river orders: χ^2 =^ 46.333, p-value = 7.5e-08). Furthermore, *G. roeselii* was on average found in more gently sloping reaches (see above) than *G. fossarum* (mean 4.1%, median 1.2%, max 58.8%; Wilcox test: W = 69009, p-value = 2.2e-16; Fig. 2c).

**Fig. 3:**
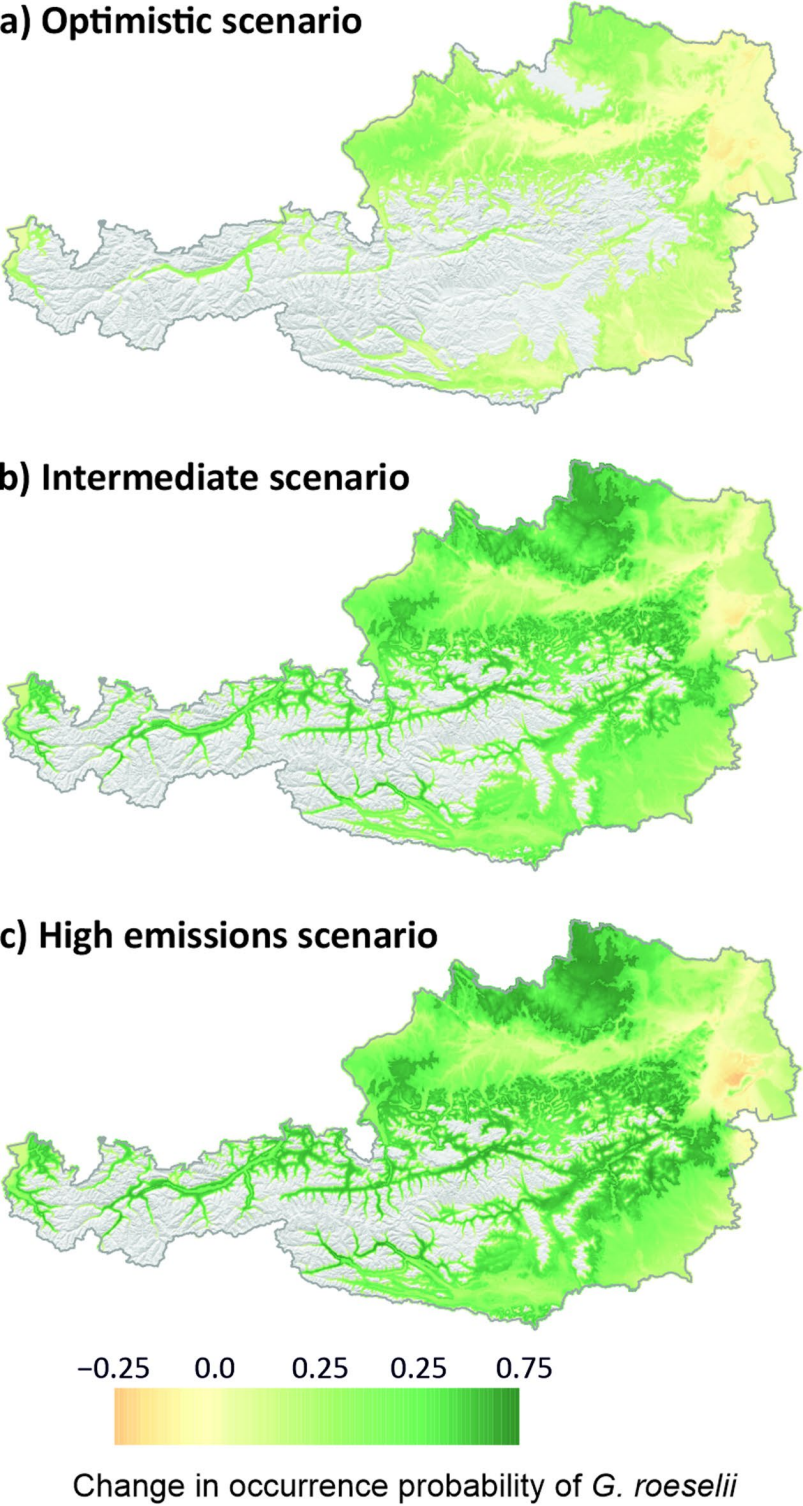
Change in occurrence probability of *G. roeselii* in Austria under optimistic (**a**), intermediate (**b**), and high emissions climate scenarios (**c**) for 2071–2100. Green: increase in probability of occurrence of G. *roeselii*; yellow: no change; orange: slight decrease; grey: no current and no predicted future occurrence. Maps were generated using R 4.4.3.

Ensemble species distribution modelling (SDM; Fig. 3) revealed comparable contributions of the four included bioclimate variables to the current distribution of *G. roeselii*. The largest contributor was Mean Temperature of Warmest Quarter (36.34%), followed by Annual Precipitation (24.33%), Temperature Seasonality (19.95%) and Precipitation Seasonality (19.37%). Model evaluation metrics are reported in Supplementary Tables S4 and S5. Projections of the distribution of *G. roeselii* under future climate scenarios for the period 2071–2100 predicted range expansions and an increased probabilities of *G. roeselii* occurrence especially in lowlands and alpine valleys (Fig. 3). In particular, the probability of occurrence in areas, where *G. roeselii* is currently absent, increases substantially with worsening climate scenarios.

### Genetic diversity of *G. roeselii* and its expansion in Central-Western Europe

**Fig. 4:**
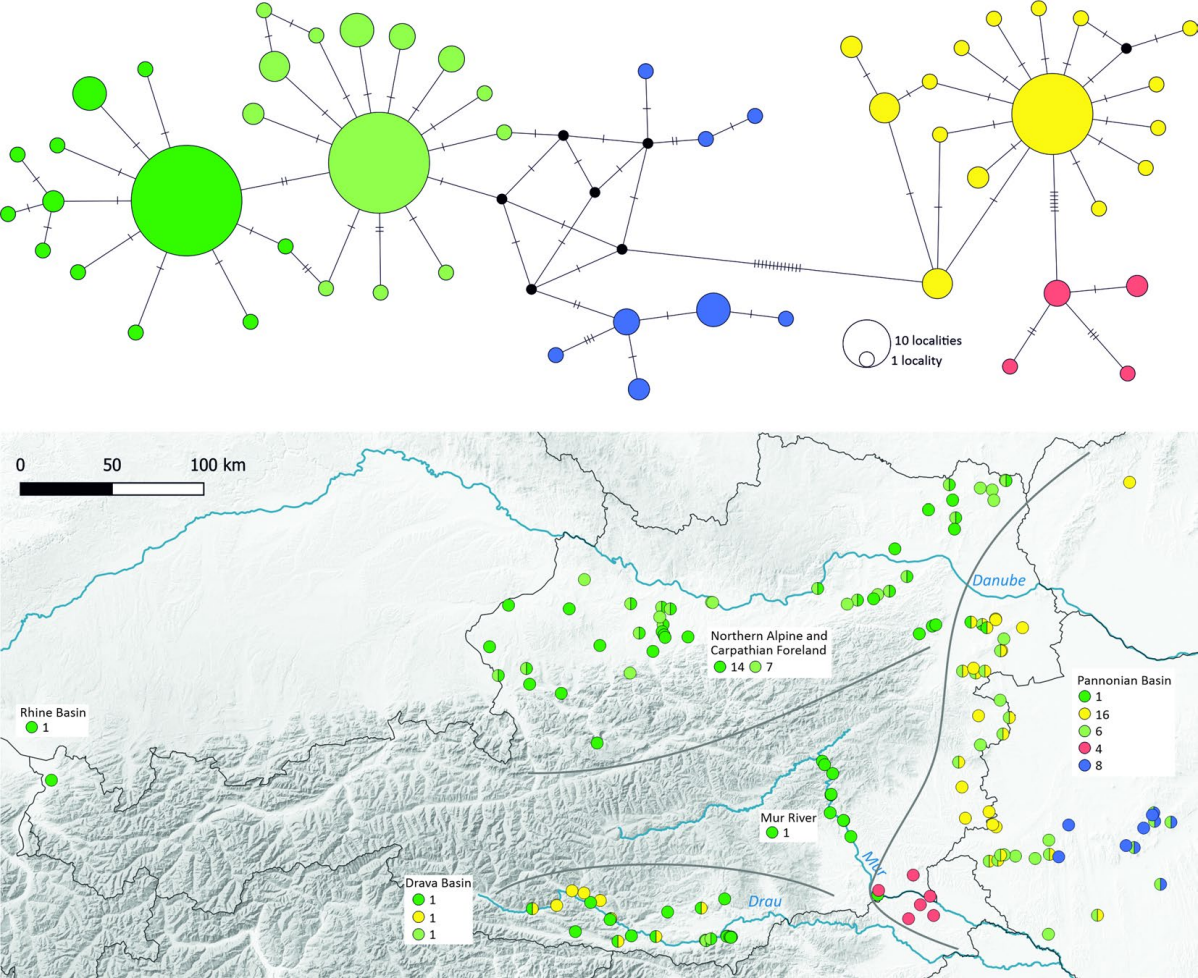
Upper: Median Joining haplotype network, based on a 658 bp long fragment of COI. Colours distinguish groups of haplotypes. Only one sequence per site and haplotype was included in the network, such that the size of the circles represents the number of sites at which a given haplotype was detected (avoiding biases introduced by sample size differences across sites). See supplementary Fig. S3 for a network including all sequenced individuals. Lower: The distribution of the haplotype groups in the study area (colours correspond to the network). Grey lines delimit the four geographic regions, which are identified in the inset boxes. The boxes also report the numbers of different haplotypes per haplotype group in the respective geographic region. Map was generated using Q-GIS 3.36.0 (http://www.qgis.org).

DNA barcode sequences (658 bp of the COI gene) were obtained from 528 *G. roeselii* individuals. The sequences were used to assign the individuals to MOTUs and BINs defined in previous studies^6,16^, and to assess genetic diversity in the study region. All 519 individuals from Austria and neighbouring regions of the Pannonian Basin belonged to MOTU C, BOLD BIN AAY1309, which is widespread in western and central Europe (Supplementary Fig. S2, Supplementary Table S2). Nine individuals from eastern Hungary belonged to BINs ADA0637 and ADD6734^16^ (Supplementary Table S2). The following analyses were restricted to BOLD BIN AAY1309. To facilitate the description of patterns of genetic diversity as well as the comparison to previously described genetic structure^16^, we distinguish five groups of haplotypes in the median joining network (Fig. 4), which represent various levels of diversity and degrees of divergence from each other. The first three groups include two closely related groups (light-green and dark-green in Fig. 4), each consisting of one central and several derived haplotypes, and a rather diverse group of haplotypes (blue in Fig. 4) which differ from the ‘green’ groups by few mutations. They correspond to BIN AAY1309 of Csapo et al.^16^, and ‘light-green’ and ‘dark-green’ haplotypes are widespread across Central-Western Europe (supplementary Fig. S2). A fourth group (yellow in Fig. 4) is clearly distinct and consists of one central and several derived haplotypes. This group corresponds to the former BIN ADD6100^16^, which is now merged with BIN AAY1309. The fifth group comprises four additional haplotypes (red in Fig. 4) and corresponds to former BIN ADD4052 of Csapo et al.^16^ (now also merged with BIN AAY1309).

The highest genetic diversity was found in the Pannonian Basin, where we detected all five haplotype groups as well as multiple different haplotypes in all but one of these groups, respectively. Haplotypes of the ‘red’ and ‘blue’ groups were found only in specific areas of the Western Pannonian Basin (Fig. 4, Supplementary Fig. S2). The ‘yellow’ haplotype group was more widespread in the Western Pannonian Basin, but also occurred in the drainage of the Drava River in the southern Alps (Fig. 4, Supplementary Fig. S2). One of the widespread Central-Western European haplotype groups (light-green) was also widespread and genetically diverse in the Western Pannonian Basin, while the other (dark-green) group was narrowly distributed and represented by only one haplotype. In contrast, these two haplotype groups overlapped and co-occurred throughout the Northern Alpine and Carpathian Foreland (Fig. 4), where each of them was represented by one frequent and several derived haplotypes. *Gammarus roeselii* collected in far western Austria (Rhine drainage) belonged to the ‘dark-green’ group (Fig. 4). In contrast to the genetic diversity existing in the Pannonian Basin and the Northern Alpine and Carpathian Foreland, *G. roeselii* in two additional regions were genetically depauperate. One of these regions is a mid-reach stretch of the River Mur, where all *G. roeselii* possessed the most frequent haplotype of the ‘dark-green’ group (Fig. 4). The other region with reduced genetic diversity belongs to the Drava Basin. There, three haplotypes were detected which represented the most frequent haplotypes of the ‘light-green’, ‘dark-green’ and ‘yellow’ groups, respectively (Fig. 4).

The star-shaped topologies of the ‘light-green’, the ‘dark-green’ and the ‘yellow’ haplotype group (Fig. 4; Supplementary Fig. S3), as well as mismatch distributions, Tajima’s D and Fu’s F (Table 1) indicate past demographic/spatial expansions of these haplotype groups. The network topologies and expansion statistics are not affected by the exclusion of the genetically depauperate Mur and Drava Basin populations (Table 1, Supplementary Fig. S4). The estimated τ parameters of the mismatch distributions (Table 1), which scale with the time of onset of the expansion, are similar to estimates in Csapo et al.^16^ (τ = 2–4 for AAY1309, our ‘light-green’ and ‘dark-green’ groups; and τ = 0.77 for ADD6100, our ‘yellow’ group), suggesting that the expansions indicated for our study region were part of the post-glacial expansion of *G. roeselii* into Central-Western Europe^16^. In the Western Pannonian Basin, expansion signals are strong for the ‘yellow’ group, but less pronounced for the western Pannonian ‘green’ group (‘light-green’ haplotypes plus one ‘dark-green’ haplotype; Table 1).

**Table 1:** Results of mismatch distribution analyses and Tajima’s D and Fu’s F neutrality tests for different subsets of samples from ‘green’ (pooled sample of ‘light-green’ and ‘dark-green’ haplotypes) and ‘yellow’ haplotype groups (see Fig. 4). SSD = Sum of Squared Deviation, Hr = Harpending’s Raggedness.

Demographic expansion	Green group			Yellow group	
	All samples	Without Mur and Drava	Pannonian only	All samples	Pannonian only
τ	2.461	2.153	0.016	1.379	1.18
SSD (p-value)	0.041 (0.141)	0.012 (0.487)	0.341 (0)	0.001 (0.834)	0.001 (0.792)
Hr index (p-value)	0.143 (0.099)	0.068 (0.466)	0.093 (1)	0.101 (0.613)	0.053 (0.651)
Spatial expansion					
τ	2.148	2.365	1.327	0.92	0.955
SSD	0.026 (0.387)	0.018 (0.205)	0.003 (0.712)	0 (0.722)	0 (0.771)
Hr index (p-value)	0.143 (0.413)	0.069 (0.191)	0.093 (0.803)	0.101 (0.8)	0.053 (0.8)
Tajima’s D (p-value)	−1.562 (0.024)	−1.527 (0.04)	−1.437 (0.053)	−1.869 (0.005)	−1.789 (0.009)
Fu’s F (p-value)	−15.291 (0)	−14.771 (0)	−3.181 (0.057)	−15.667 (0)	−14.173 (0)

**Fig. 5:**
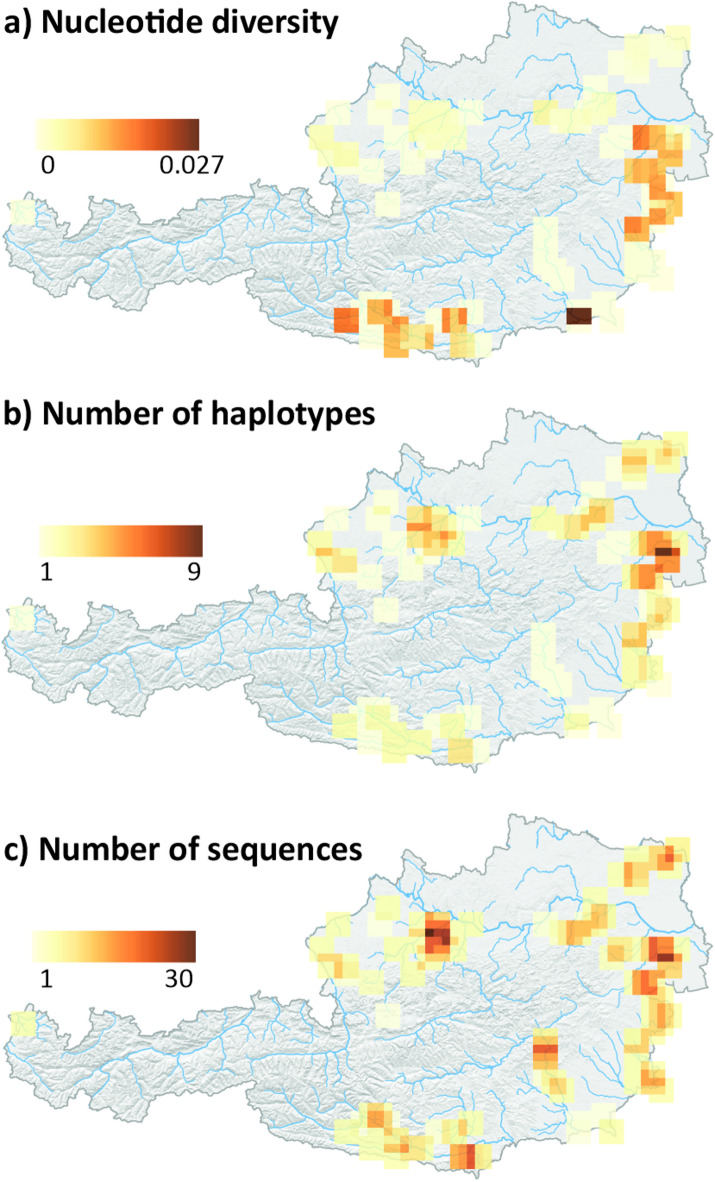
Nucleotide diversity, number of haplotypes, and number of sequences of *G. roeselii* sampled across Austria. Values for each 6.7 km wide cell were calculated from individuals present in this and all neighbouring cells (approximately 20 km x 20 km). Maps were generated using R 4.4.3.

To visualize the spatial distribution of genetic diversity across Austria, we used a sliding window approach and calculated nucleotide diversity, number of haplotypes and sample size in sliding windows of 20 × 20 km across Austria (Fig. 5). As nucleotide diversity reflects the average divergence between COI sequences (Fig. 5a), it was highest in areas where multiple haplotype groups co-occur, i.e., in the Pannonian Basin, the Drava Basin and in a small area where haplotypes of the western Pannonian ‘red’ groups were sampled near the river Mur, which is inhabited by a single ‘dark-green’ haplotype. In contrast, the number of haplotypes (Fig. 5b) reflects diversity both between and within haplotype groups and was highest in some parts of the Northern Alpine and Carpathian Foreland and in the Western Pannonian Basin. The distribution of sample sizes (number of individuals sequenced) across the study area (Fig. 5c) indicates that the limited genetic diversity in the Mur and the Drava Basin is not due to insufficient sampling, whereas sample size and number of haplotypes co-vary in the other regions.

## Discussion

The sampling campaign of epigean amphipods across Austria encompassed diverse landscapes from the eastern European Alps to the western Pannonian lowland. *Gammarus roeselii* was primarily found in areas surrounding the alpine region, but also in some inner-alpine valleys and lakes. Towards the west, in neighbouring Switzerland, the distribution of *G. roeselii* is restricted to Lake Constance and the lowest reaches of its tributary streams ^44^as well as the Rhine and the Aare rivers, but the species is absent from other waterbodies in the Swiss alpine foreland^45^. The ranges of *G. roeselii* in the northern and eastern parts of Austria are connected to the distributions of the species in Central-Western Europe and the Pannonian Basin, respectively (Fig. 1). The occurrence of *G. roeselii* in the lotic waterbodies of Austria is correlated with gentle stream slope (a proxy for slow flow velocity), low elevation and high ambient temperature in the warmest quarter. In the study area, these environmental variables are also correlated with each other, which makes it difficult to identify the decisive factor that facilitates the occurrence of *G. roeselii*. Most lotic waterbodies of Austria, especially outside the Pannonian Basin, are inhabited by *G. fossarum*, which puts *G. roeselii* in competition with *G. fossarum*. Temperature is likely playing a major role in determining the relative competitive advantages of the two species, as *G. roeselii* develops faster and has higher fecundity than *G. fossarum* in warmer water^27,31^. Additionally, flow velocity might limit the distribution of *G. roeselii* regardless of temperature, as *G. roeselii* have been shown to be susceptible to displacement in the drift^26^ and to suffer adverse effects of hydrodynamic forces on growth and energy storage^46^. Furthermore, *G. roeselii* prefers biotic over lithal (rocky or stony) substrate^24,47^, and the predominance of gravel and cobbles in headwaters and streams in mountainous regions may restrict its ability to compete with *G. fossarum*. The differentiation of the habitat preferences between *G. roeselii* and *G. fossarum* in our study region matches the generally observed longitudinal gradient of the two species in running waters: *Gammarus fossarum* preferably inhabits springs and upstream reaches, while *G. roeselii* is found in the lower reaches of rivers and streams^24,31,36,44^. We acknowledge the coarse scale of the environmental parameters used in this study, as our focus on sampling density came at the expense of the on-site documentation of environmental parameters. Data on flow velocity, temperature, oxygen, type of substrate, etc. would perhaps give additional insights into habitat preferences and differences between studied species, although we note that the temporal variability of these variables might also diminish the relevance of measurements taken at the time of sampling.

Projections under future climate scenarios predicted substantial expansions of suitable areas for *G. roeselii* in the near future, especially in the lowlands and pre-alpine regions, and along large river valleys. To address the uncertainty in the future climate projections, we used three different climate models reflecting different emission scenarios. While modelling under the low emissions scenario predicted only a slight range expansion of *G. roeselii*, the predicted expansion of *G. roeselii* under the higher emission scenarios extended substantially into the range that is currently inhabited by *G. fossarum*, potentially entailing substantial pressure on the native *G. fossarum*^25,48,49^. The prediction models included bioclimate variables on a coarse scale, ignoring fine-scale habitat properties (e.g., flow velocity), and should therefore be viewed as coarse approximations. Importantly, physical and chemical anthropogenic disturbances of streams and rivers may further promote the spread of *G. roeselii*, while negatively affecting *G. fossarum*. *Gammarus roeselii* exhibit a high tolerance to ammonia^50^ and other pollutants^25,48,51^, and benefit from warm and more lentic conditions imposed by river damming^52^.

Spreads of lacustrine or eurytopic species into the artificial lentic habitat of dammed rivers, and an associated advance of alien species, have already been observed in fish communities across the world^53^. The colonization of the river Mur by *G. roeselii*, as found in this study, may already have been promoted by anthropogenic alterations of river morphology. The approximately 100 km mid-stretch of the river along which we found *G. roeselii* lies in hilly landscape between the towns of Bruck an der Mur and Spielfeld and is characterized by a chain of hydropower dams generating potamon to lacustrine conditions along much of its length. Notably, in this section, *G. roeselii* were detected only in the main river but not in the tributaries, which are inhabited by *G. fossarum*. Conversely, the adjacent downstream reach of the Mur is free-flowing with a rhithron character, and there, no *G. roeselii* were found in the main river while they occurred in some of its tributaries. Even further downstream, near the confluence with the River Drava in Croatia, *G. roeselii* constituted only 0.1% of macroinvertebrates in the river Mur while *G. fossarum* were common (74%^54^). Moreover, repeated faunistic surveys conducted between 1977 and 1979 (pre-dating some of the current hydropower dams) did not reveal any *G. roeselii* in the aforementioned mid-stretch of the River Mur, where *G. roeselii* is now common^55^. A recent origin of the Austrian mid-stretch Mur population is also suggested by the absence of genetic diversity at the COI locus, as all individuals carried the same high-frequency haplotype of the ‘dark-green’ group. In contrast, *G. roeselii* collected from waterbodies around the adjacent free-flowing reach of the Mur, which is already part of the Western Pannonian Basin, belonged to a different haplotype group and displayed considerable genetic diversity.

Genetic diversity is also remarkably low in *G. roeselii* inhabiting various waterbodies in the Drava Basin, suggesting that the area was likewise only recently colonized. The presence of three COI haplotypes, which are also common elsewhere (two with a Central-Western European and one with a western Pannonian distribution), indicate at least three successful colonizations. Colonization of the area may have been facilitated by the damming of the lower reaches of the river Drava, but currently, *G. roeselii* also occur elsewhere in the region in slow-flowing and summer-warm waterbodies, while they face competition by invasive amphipods in the dammed sections of the Drava (^54^ and own data). The occurrences of *G. roeselii* in the Drava Basin and the Mur River are geographically isolated from the remaining distribution ranges of the same haplotypes and may be due to human-mediated introductions, possibly along with allochthonous fish stocks. Fish farms in the Drava area obtain some of their stocks from hatcheries located in the Northern Alpine Foreland, which is also visible in the genetic composition of fish south of the Alps^56^. In the Drava Basin, the ‘light-green’ *G. roeselii* haplotype is restricted to two adjacent locations near a place where we also found an introduced *G. fossarum* MOTU, which is otherwise distributed in the Northern Alpine Foreland (unpublished data).

Alternatively, *G. roeselii* may have colonized the Mur and the Drava Basin in the course of its postglacial expansion across Central-Western Europe, and its genetic diversity may have been lost in recent population bottlenecks. However, we know of no geologic, climatic or anthropogenic disturbances, which would recently have decimated (rather than promoted) *G. roeselii* in the regions concerned. Although invasive gammarids (*Dikerogammarus haemobaphes*) currently outcompete *G. roeselii* in the lower reaches of the Drava, this is not the case in the region where we sampled the majority of the Drava Basin *G. roeselii*, and no invasive amphipods have yet been detected in the Mur (despite extensive sampling; own unpublished data). We therefore consider a recent colonization of the Drava Basin and the mid-stretches of the Mur River as the most likely explanation for the low genetic diversity of *G. roeselii* in these regions.

While the expansion of *G. roeselii* into headwater and mountainous water bodies of Central-Western Europe appears to be in progress (our data and^15,21^, the species is losing ground to invasive amphipods in potamal and lacustrine habitat^12,30^. For example, although *G. roeselii* was present in the mainstream of the Danube until the 1950 s, it was already reported as rare in the 1990s^36^, and not found in the Danube at all in recent studies^57^. Its disappearance from the Danube might be connected with the invasion of several Ponto-Caspian amphipod species, like *Dikerogammarus villosus*, *D. haemobaphes*, and *Chaetogammarus ischnus*, that were first found in the Danube in the late 1980s^36^. In the lower, dammed reaches of the river Drava in Croatia, *D. villosus* is currently more abundant than *G. roeselii*^54^. As aggressive invader, *D. villosus* has many advantageous behavioural, physiological, and life history traits that contribute to its replacement of native species in rivers^12,46,58^.

Our study region lies at the geographic border between the proposed Pannonian glacial refugium and the post-glacial expansion range into Central-Western Europe^16^. While all COI haplotypes detected in the region belong to the same genetic cluster (BOLD BIN), patterns of haplotype diversity vary regionally. The Western Pannonian Basin harbors the highest diversity within the study area, both in terms of the number of haplotypes and the haplotype groups. Phylogenetic diversity is even higher in north-eastern, eastern and southern parts of the Pannonian Basin, where several distinct BOLD BINs (all within MOTU C) were recorded^16^ (see also Supplementary Fig. S2). In the present study, two groups of haplotypes showed signals of population expansion in the Western Pannonian Basin. The expansion of the ‘green’ haplotype group is part of the large-scale, post-glacial colonization of *G. roeselii* across Central-Western Europe^16^ (Supplementary Fig. S2). Restricting the analysis to the western Pannonian ‘light-green’ haplotypes further increases the strength of the expansion signal (not shown). In contrast, the expansion of the ‘yellow’ haplotype group remained restricted to the western Pannonian, except for occurrences in the southern Alps (Drava Basin; discussed above) and isolated sites in the Netherlands and Italy (Supplementary Fig. S2). The genetic diversity of *G. roeselii* in the Northern Alpine and Carpathian Foreland reflects the genetic diversity found across Central-Western Europe, indicating that this region belongs to the post-glacial expansion range^16^. The widespread Central-Western haplotypes are closely related to a group of genetically diverse haplotypes (the ‘blue’ group) restricted to the South-Western Pannonian Basin, which suggests that this region is the source of the post-glacial expansion across Central-Western Europe. Nuclear markers (for example fast-evolving ITS sequences, or genome-wide SNP data) would be beneficial to validate the COI-sequence based inferences and generate additional insights into the demographic history of *G. roeselii* in the area.

To summarize, the genetic diversity patterns indicate that *G. roeselii* are native to a large part of the study region. However, both ecological and genetic data also indicate ongoing changes in the distribution of *G. roeselii*, with the rate of change potentially being accelerated by anthropogenic and climatic influences. While its preferences for warmer temperature and its robustness against anthropogenic stressors give *G. roeselii* an advantage over the more susceptible *G. fossarum*, there is evidence that *G. roeselii* may be under pressure from non-native amphipods that are even better adapted to deteriorating river habitats. Future monitoring efforts therefore need to consider the interactions between these various species, while management actions need to ensure the preservation of pristine and diverse lotic habitats in order to support native species.

## Methods

### Sampling

Sampling of *G. roeselii* and other amphipods was conducted between 2022 and 2025 and covered 1003 sites in Austria and adjacent regions. Amphipods were collected by disturbing the stream bed and detritus patches, or the lake bottom, upon which the animals were flushed into hand nets. The sampling procedure was not standardized across sites, but adjusted to local conditions such as stream bed structure and amphipod abundance, aiming at sample sizes of at least 50 individuals if possible. All collected amphipods were identified morphologically using a stereomicroscope and stored in 99% EtOH. Up to five individuals of *G. roeselii* per site were selected for DNA sequencing (see below).

### Habitat analyses

Given the limited geographical range and sample sizes in the Elbe basin in the far north of Austria and in the Rhine basin in the far west (only two sites with *G. roeselii*, 25 sampled sites altogether), we limited the analysis of habitat preferences to the sites in the Danube basin in which we had found *G. roeselii* and/or *G. fossarum*, as well as sites without amphipods (917 sites). Eleven of those sites were standing waters. For the remaining 906 sites, we gathered information on the type of habitat (stream/standing water/spring). Stream and spring sites were characterized by three stream properties: river order (eight categories: spring and orders from 1 to 7), the drainage area (five categories: spring, < 10 km^2^, 10–100 km^2^, 100–1000 km^2^, and > 1000 km^2^), and slope of the stream, as a proxy for flow velocity. We used Austrian federal river network data as a basic source (https://www.data.gv.at/katalog/dataset/c2287ccb-f44c-48 cd-bf7c-ac107b771246). As the official river network layer does not include all small streams, we manually added missing streams to the network, and corrected it so that all branches were connected. We calculated the slope of the sections above and below the sampling sites as follows: for each site, we determined section end points on the stream 100 m above and below our site. If a branching (confluence) occurred less than 100 m away from the sampling site in one direction, we only used the other section end point. If confluence occurred less than 100 m away in both directions, we selected the branching nodes as our end point and calculated their distance from the sampling site. Sites close to the source (spring) had only downstream end points. We inferred the elevation of the site and of the section end points from the Austrian Digital Elevation Model (https://www.data.gv.at/katalog/dataset/land-ktn_digitales-gelandemodell-dgm-osterreich), with a resolution of 10 m and elevation values rounded to 1 m. We calculated the slope as the difference in elevations/distance between sites and section end points. For sites with two end points, we used the lower of the two slope values for the analyses. We evaluated the obtained slopes and manually corrected erroneous values (large sinks/hills). We decided on this approach as the widely used modelling of the river network and slope from terrain data was not feasible for such a large area, and with the majority of sampled sites being in small streams. Next, we assigned the river order to each site according to Bundesberichtsgewässernetz GGNv15 (https://www.data.gv.at/katalog/dataset/e73a9425-a833-4579-9e6e-3ca76a1ea943). For streams not included in the official river network, we inferred river order values from the corrected river network (this concerned mainly first-order streams). Artificial side-channels were assigned the value of the stream from which they were branching. Drainage size category was inferred from Gesamtgewässernetz - Fliessgewässer (Routen) (https://www.data.gv.at/katalog/dataset/c2287ccb-f44c-48 cd-bf7c-ac107b771246).

Additionally, sampling sites were characterized by elevation (see above) and by mean temperature of warmest quarter in period from 1981 to 2010 (bio10) obtained from the CHELSA portal (https://chelsa-climate.org/bioclim/)^59,60^.

The frequencies and distributions of sampling sites across the categorical and continuous habitat variables were illustrated by stacked bar plots and boxplots, respectively. To test whether *G. roeselii* occupy sites independent of the habitat variables ‘river order’ and ‘drainage size’, we compared the observed frequencies of sites inhabited by *G. roeselii* in the different categories of river order and drainage size to random subsets drawn from all sites. Specifically, we randomly sampled 1000 subsets of 133 sites (number of sites with *G. roeselii* in lotic waterbodies in the Danube basin) from all 906 sites. We compared the observed frequencies of sites within the variable categories to the random distributions, deriving p-values from the proportion of random subsets that yielded values that were more extreme (higher or lower) than the observed ones. In the same way, we tested for an effect of stream slope on the occurrence of *G. roeselii*. We determined minimum, mean, median, and maximum values of stream slope in 1000 random subsets of sites and compared the distributions of the summary statistics to the observed data. Applying two-sided hypothesis testing, a threshold of *p* < 0.025 was used to assign statistical significance to the permutation tests. Next, we tested for differences in habitat variables between *G. roeselii* and *G. fossarum* sites (sites where both species were present were included in both groups). Stream slopes were compared between groups using a Wilcoxon rank sum test. Differences in the categorical variables were investigated using Pearson’s Chi-squared test with a Monte Carlo simulation for p-value computation. Elevation and temperature data were not analysed statistically because of the large geographical component in their variance.

We used Q-GIS 3.36.0 (http://www.qgis.org) for data preparation, and R 4.4.3^61^ for data preparation, plotting, and statistical analyses.

### Species distribution modelling (SDM)

Ensemble SDM approach was used to first model *G. roeselii* distribution under contemporary climatic conditions, and then to predict future distribution under various climate scenarios.

We downloaded all 19 bioclimatic variables with 1 km resolution as mean values for period from 1981 to 2010 from the CHELSA portal (https://www.chelsa-climate.org/datasets/chelsa_bioclim)^59,60^ and cropped them to Austria. We tested for correlations between them using the Spearman’s correlation coefficient and the variance inflation factor (VIF)^62^. Variables were grouped into five clusters based on correlation coefficients with cut-out value of 0.7. Next, we calculated VIF. We selected four variables (representing four of the clusters, and having VIF below 3) to use in SDM: Mean Temperature of Warmest Quarter (bio10), Temperature Seasonality (bio04), Annual Precipitation (bio12), and Precipitation Seasonality (Coefficient of Variation) (bio15). The fifth cluster was represented by Isothermality and was not included in the analysis due to its low explanatory power in preliminary analyses. We kept the default raster resolution of 30 arc seconds (app. 681 m).

We used the same dataset of presences and absences of *G. roeselii* (906 sites; 133 presences, 773 absences) as in the above analysis of habitat preferences. To account for spatial autocorrelation, we spatially thinned the dataset as follows: first, we removed 16 absences less than 2 km (three times the width of the raster cell) away from presences; next, we thinned the dataset to a minimum distance of 2 km between presences (removed 25 sites), and 2 km between absences (removed 218), using a randomisation approach (package *spThin*^63^. The final number used for modelling was 567 absences and 112 presences.

We used the *SSDM* package^64^, with an ensemble SDM approach (function *ensemble_modelling*), where SDM outputs of different modelling algorithms are combined, weighted by the chosen metric and averaged into a consensus model. Models were run using eight algorithms, that were trained and tested using the default parameters: Generalized linear model (GLM), Generalized additive model (GAM), Multivariate adaptive regression splines (MARS), Classification tree analysis (CTA), Random Forest (RF), Maximum entropy (MAXENT), Artificial neural network (ANN), and Support vector machines (SVM). Each model was run in 10 iterations. The area under the ROC curve (AUC) value of 0.8 was used to calculate the threshold to include and weight the individual models into an ensemble model. Models were evaluated with the tools integrated in the SSDM package, and evaluation metrics of algorithms that passed the inclusion threshold, as well as correlations between alternative statistical methods, are reported in Supplementary Tables S4 and S5. Relative contributions of the climate variables were assessed based on Pearson’s correlation coefficient.

To investigate impacts of changing environmental conditions on the distribution of *G. roeselii* in Austria, we projected the ensemble model with environmental conditions under three future climate scenarios for 2071–2100, using GFDL-ESM4 climate models: optimistic (SSP1-RCP2.6), intermediate (SSP3-RCP7.0), and high-emissions scenario (SSP5-RCP8.5)^59,60^. From each projection, we took the resulting rasters of probability of occurrence in space and rounded low probabilities (< 0.1) to zero for plotting purposes. Next, we subtracted the contemporary raster of probabilities from each of the three projections to compare the change in the probability of occurrence of *G. roeselii* across Austria in the future. The analysis was done in R 4.4.3^61^.

### Genetic data generation

DNA was extracted from sample tissue (typically a piece of a leg) using a standard Chelex protocol^65^. The COI barcode marker was amplified using primers LCO1490-JJ2 and HCO2198-JJ2^66^, using the polymerase chain reaction (PCR) protocol described in^67^. For 10 individuals, Sanger sequencing was carried out as described in^68^. The majority of amplicons (from 509 individuals) were sequenced using Oxford Nanopore Technology (ONT) on Flongle flow cells (versions R9.4.1; FLO-FLG001 and R10.4.1; FLO-FLG114), and basecalling of raw reads was carried out with Guppy version 6.4.2-1.2. Details on the ONT sequencing and basecalling protocols are given in^67^. Demultiplexing of reads and COI barcode calling was carried out using the ONTbarcoder software^69^ with default settings^67–69^. Nanopore-based COI sequences were shown to be of the same quality and consistent with Sanger sequencing^68^ (own data). In total, we obtained complete 658 bp COI barcode sequences from 528 individuals.

### Genetic analyses

We gathered all available genetic information on *G. roeselii* from public databases (GenBank and Bold, accessed in June 2024). We aligned newly obtained and published sequences of the COI marker using MEGA 11.0.13^70^, and used ASAP (Assemble Species by Automatic Partitioning)^71^ to delimit MOTUs^6,16^. Next, we searched our sequences in BOLD (https://boldsystems.org/), where every sequence was assigned to an existing Barcode Index Number (BIN)^16^.

We inferred median-joining networks in PopArt 1.7^72^, with the homoplasy level set to default value (ε = 0). First, we restricted the analysis to the newly generated 658 bp sequences, and generated three networks: one network including only one individual per haplotype and site (illustrating the number of sites at which the various haplotypes were detected), one including all individuals (illustrating the number of individuals carrying the different haplotypes), and one including all individuals except those from Drava Basin and Mur River (to exclude the populations of putatively very recent origin). We used Q-GIS 3.36.0 (http://www.qgis.org) to plot the distribution of haplotype groups, which were identified in the networks, on a map.

Next, we also inferred a haplotype network including both own and published data. This was done separately, as many of the published sequences were shorter and the alignment had to be pruned to 519 bp.

We analysed historical demographic and spatial expansion using mismatch distribution analyses, Tajima’s D^73^, and Fu’s Fs^74^ neutrality tests in Arlequin 3.5.1.3^75^. Statistical significance of the estimated parameters was assessed based on 1000 bootstrap replicates. Expansion tests were carried out on five different subsets of samples selected based on haplotype group and region of origin, as described in the Results section^75^.

Finally, we illustrated spatial patterns of genetic diversity in *G. roeselii*. To this aim, we laid a grid of 6.7 km x 6.7 km cells over Austria and used a sliding window approach to plot a continuous map of diversity and avoid the impact of randomly placing the grid on the map. Using the set of 658 bp sequences obtained in the current study, each cell was assigned a nucleotide diversity value (sum of the number of differences between pairs of sequences divided by the number of comparisons (Nei 1987)) based on the samples that fell within the cell and all its neighbours (amounting to an approximately 20 km x 20 km window). Numbers of different haplotypes and sample sizes (numbers of sequenced individuals) across space were plotted in the same manner. Calculations were carried out using R 4.4.3^61^, and package *pegas*^76^.

### Maps

All maps were created using publicly available data. Hillshade was generated using get_elev_raster() function from *elevatr* package, using Amazon Web Services Terrain Tiles, an open source global elevation dataset (https://registry.opendata.aws/terrain-tiles/). Rivers were depicted using Austrian Water Network data (Bundesberichtsgewässernetz GGNv15 (https://www.data.gv.at/katalog/dataset/e73a9425-a833-4579-9e6e-3ca76a1ea943). Polygons of country borders were downloaded from Eurostat webpage (https://ec.europa.eu/eurostat/web/gisco/geodata/administrative-units/countries).

## Supplementary Information

Below is the link to the electronic supplementary material.

Supplementary Material 1

Supplementary Material 2

## Data Availability

Sequences were deposited at GenBank under accession numbers PX314984 - PX315511. All data and code used to produce the paper is available at figshare (https://doi.org/10.6084/m9.figshare.31123501).
