# Supplementary material for: Habitat preferences and genetic diversity of the amphipod Gammarus roeselii across the Eastern Alps and western Pannonian Basin
Source: Sci Rep. 2026 Feb 13;16:8607. doi: 10.1038/s41598-026-39958-7 (PMC12976119; doi:10.1038/s41598-026-39958-7)
Supplement: Supplementary file 1 — Supplementary Material 1 [file 41598_2026_39958_MOESM1_ESM.docx]

**Supplementary Information**

**Habitat preferences and genetic diversity of the amphipod *Gammarus roeselii* across the Eastern Alps and western Pannonian Basin**

Špela Di Batista Borko^1^, Jacqueline Grimm^1^, Christoph Hahn^1^, Péter Takács^2^, Anna-Maria Greilberger^1^, Stephan Koblmüller^1^ & Kristina M. Sefc^1^

^1^ Institute of Biology, University of Graz, Graz, Austria

^2^ HUN-REN Balaton Limnological Research Institute, Tihany, Hungary

**Supplementary Tables**

**Table S1:** Sampled sites. Each site has information on geographic position and coordinates, elevation, collection date, sampled species, and three habitat variables: river order, drainage and slope upstream and downstream from site. Provided as separate excel document.

**Table S2:** Sequenced individuals, together with ID, GenBank accession number, BIN, and ID of the sample site (ID_locality). ID_locality corresponds to sample site data in table S1. Provided as separate excel document.

**Table S3:** Tests for habitat preferences. The table shows observed count values (number of *G. roeselii* presence sites) per drainage size and river order category, and the summary statistics of stream slope at *G. roeselii* presence sites. The p-values (two-sided) were obtained from permutations testing for random distribution of *G. roeselii* across all sampling sites. To account for multiple testing (16 tests), p-values were corrected according to the procedure by Benjamini and Hochberg (1995).

| **Drainage** | **Category** | **Observed count** | **p-value** | **p-value (corr.)** |
| --- | --- | --- | --- | --- |
|  | spring | 0 | 0.05 | 0.08 |
|  | < 10km² | 23 | 0 | 0 |
|  | 10-100km² | 31 | 0.339 | 0.417 |
|  | 100-1000km² | 43 | 0 | 0 |
|  | >1000km² | 36 | 0 | 0 |
| **River Order** | **Category** | **Observed count** | **p-value** |  |
|  | spring | 0 | 0.063 | 0.092 |
|  | 1 | 22 | 0.192 | 0.256 |
|  | 2 | 13 | 0.003 | 0.006 |
|  | 3 | 29 | 0.552 | 0.631 |
|  | 4 | 20 | 0.602 | 0.642 |
|  | 5 | 16 | 0.01 | 0.018 |
|  | 6 | 7 | 1 | 1 |
|  | 7 | 26 | 0 | 0 |
| **Slope** | **Statistic** | **Observed value** | **p-value** |  |
|  | mean | 0.00505 | 0 | 0 |
|  | median | 0.002 | 0.001 | 0.002 |
|  | max | 0.041 | 0 | 0 |

**Table S4:** Evaluation of the ensemble SDM models, as implemented in the SSDM package (Schmitt et al. 2017).

| **SDM ensemble** | **threshold** | **AUC** | **omission rate** | **sensitivity** | **specificity** | **prop. correct** | **kappa** | **calibration** | **kept**  **model** |
| --- | --- | --- | --- | --- | --- | --- | --- | --- | --- |
| **GLM** | 0.23 | 0.83 | 0.24 | 0.76 | 0.76 | 0.76 | 0.37 | 0.76 | 7 |
| **GAM** | 0.21 | 0.83 | 0.28 | 0.72 | 0.72 | 0.72 | 0.30 | 0.75 | 9 |
| **MARS** | 0.20 | 0.83 | 0.26 | 0.74 | 0.74 | 0.74 | 0.34 | 0.70 | 4 |
| **RF** | 0.17 | 0.85 | 0.24 | 0.76 | 0.76 | 0.76 | 0.38 | 0.71 | 10 |
| **SVM** | 0.00 | 0.82 | 0.23 | 0.77 | 0.77 | 0.77 | 0.40 | 0.31 | 4 |
| **MAXENT** | 0.48 | 0.85 | 0.46 | 0.83 | 0.52 | 0.54 | 0.12 | 0.43 | 9 |
| **ANN** | did not pass the AUC threshold | | | | | | | | |
| **CTA** | did not pass the AUC threshold | | | | | | | | |

**Table S5:** Agreement between the alternative statistical methods (Pearson’s correlation coefficients).

|  | **GLM** | **GAM** | **MARS** | **RF** | **SVM** | **MAXENT** |
| --- | --- | --- | --- | --- | --- | --- |
| **GLM** |  | 0.84 | 0.74 | 0.71 | 0.58 | 0.90 |
| **GAM** | 0.84 |  | 0.86 | 0.84 | 0.71 | 0.94 |
| **MARS** | 0.74 | 0.86 |  | 0.88 | 0.75 | 0.87 |
| **RF** | 0.71 | 0.84 | 0.88 |  | 0.77 | 0.86 |
| **SVM** | 0.58 | 0.71 | 0.75 | 0.77 |  | 0.70 |
| **MAXENT** | 0.90 | 0.94 | 0.87 | 0.86 | 0.70 |  |

Benjamini, Y., & Hochberg, Y. (1995). Controlling the false discovery rate: a practical and powerful approach to multiple testing. Journal of the Royal statistical society: series B (Methodological), 57(1), 289-300.

Schmitt, S., Pouteau, R., Justeau, D., de Boissieu, F., & Birnbaum, P. (2017). ssdm: An r package to predict distribution of species richness and composition based on stacked species distribution models. Methods in Ecology and Evolution, 8(12), 1795–1803.

**Supplementary Figures: next page**


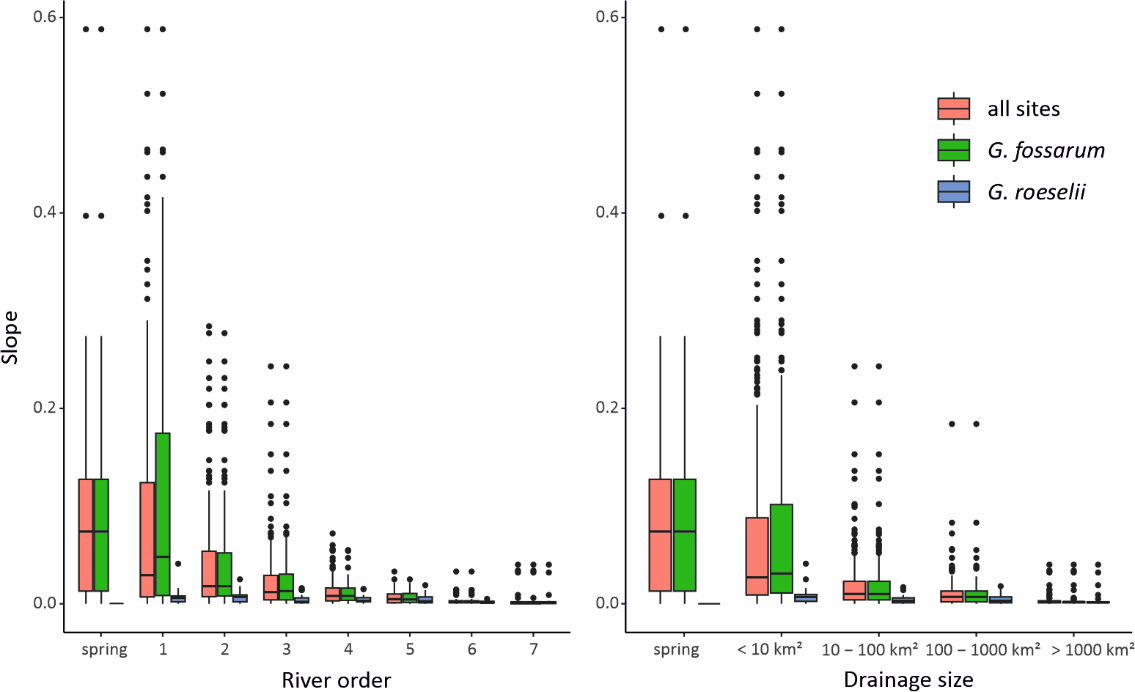


**Figure S1:** Boxplots of stream slopes for each river order (left) and drainage size (right) category, for all sites, *G. fossarum* sites, and *G. roeselii* sites separately.

**
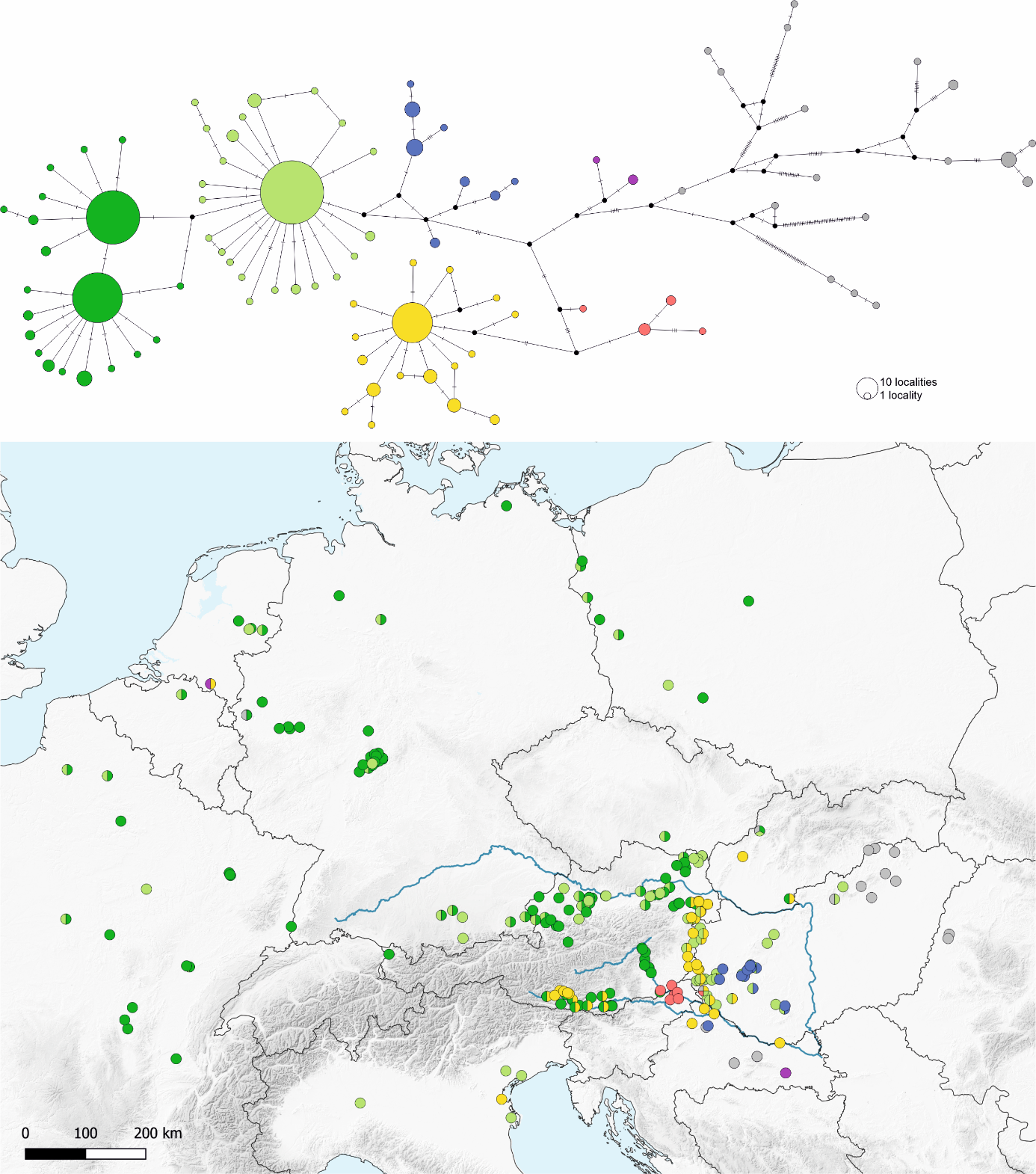
**

**Figure S2:** Upper: Median Joining haplotype network, based on a 519 bp long fragment of COI of all *G. roeselii* sequences (own and published). Focal BIN AAY1309 is coloured by groups of haplotypes. Grey samples represent other BINs (not studied here). Only one sequence per site and haplotype was included in the network, such that the size of the circles represents the number of sites at which a given haplotype was detected. Lower: The distribution of the haplotype groups of BIN AAY1309 (coloured) and other BINs (grey) in Europe. Map was generated using Q-GIS 3.36.0 (http://www.qgis.org).


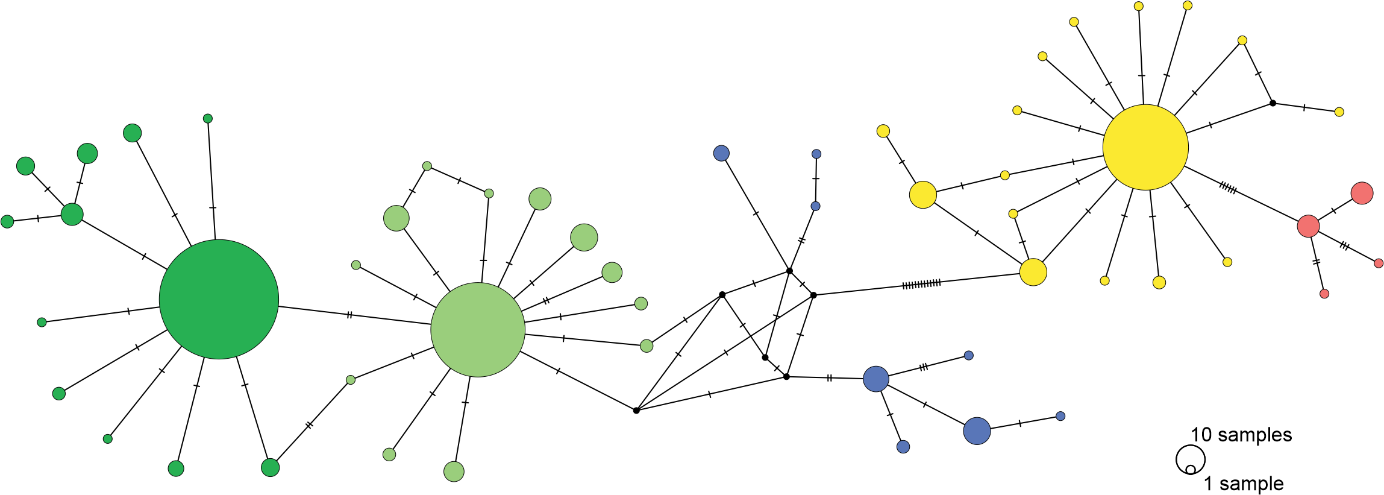


**Figure S3:** Median Joining haplotype network, based on a 658 bp long fragment of COI of all *G. roeselii* samples from the study area (519 sequences).


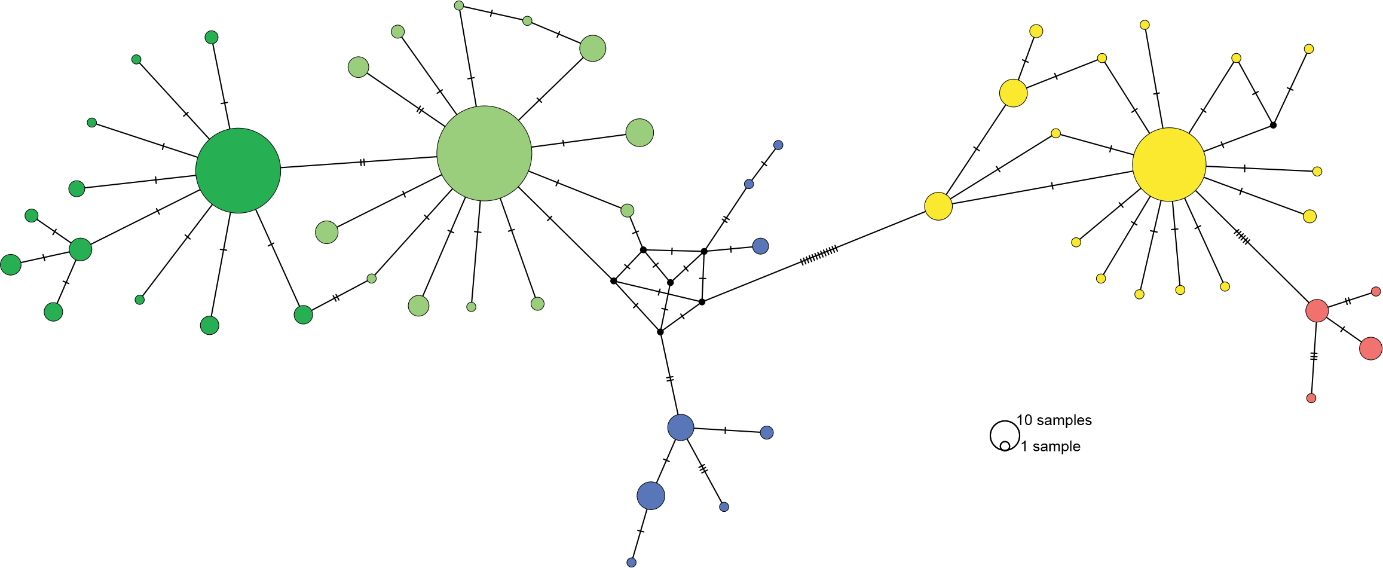


**Figure S4:** Median Joining haplotype network, based on a 658 bp long fragment of COI of all *G. roeselii* samples from the study area, but excluding samples from Mur River and Drava Basin (401 sequences).
